# Accuracy and Sterilizability of In-House Printed Patient-Specific Aortic Model for Surgeon-Modified Stent Grafts—A Workflow Description for Emergency Aortic Endovascular Procedures

**DOI:** 10.3390/jcm13051309

**Published:** 2024-02-26

**Authors:** Max Wilkat, Julian Lommen, Majeed Rana, Norbert Kübler, Tobias Wienemann, Sönke Maximilian Braß, Reinhold Thomas Ziegler, Agnesa Mazrekaj, Artis Knapsis, Hubert Schelzig, Markus Udo Wagenhäuser, Amir Arnautovic

**Affiliations:** 1 Department for Oral & Maxillofacial Surgery, Medical Faculty and University Hospital Düsseldorf, 40225 Düsseldorf, Germany; 2 Institute of Medical Microbiology and Hospital Hygiene, Medical Faculty and University Hospital Düsseldorf, 40225 Düsseldorf, Germany; 3 Department for Vascular and Endovascular Surgery, Medical Faculty and University Hospital Düsseldorf, 40225 Düsseldorf, Germany

**Keywords:** patient-specific implant, aortic reconstruction, in-house 3D-printing

## Abstract

**Introduction:** The use of 3D-printed aortic models for the creation of surgeon-modified endoprostheses represents a promising avenue in aortic surgery. By focusing on the potential impact of sterilization on model integrity and geometry, this report sheds light on the suitability of these models for creating customized endoprostheses. The study presented here aimed to investigate the safety and viability of 3D-printed aortic models in the context of sterilization processes and subsequent remodeling. **Methods:** The study involved the fabrication of 3D-printed aortic models using patient-specific imaging data and established additive manufacturing techniques. Five identical aortic models of the same patient were printed. Two models were subjected to sterilization and two to disinfection using commonly employed methods, and one model remained untreated. The models were checked by in-house quality control for deformation (heat map analyses) after the sterilization and disinfection processes. Three models (sterilized, disinfected, and untreated) were sent for ex-house (Lufthansa Technik, AG, Materials Technologies and Central Laboratory Services, Hamburg, Germany) evaluation and subsequent quantification of possible structural changes using advanced imaging and measurement technologies (macroscopic and SEM/EDX examinations). After sterilization and disinfection, each aortic model underwent sterility checks. **Results:** Based on macroscopic and SEM/EDX examinations, distinct evidence of material alterations attributed to a treatment process, such as a cleaning procedure, was not identified on the three implants. Comparative material analyses conducted via the EDX technique yield consistent results for all three implants. Disinfected and sterilized models tested negative for common pathogens. **Conclusions:** The evaluation of 3D-printed aortic models’ safety after sterilization as well as their suitability for surgeon-modified endoprostheses is a critical step toward their clinical integration. By comprehensively assessing changes in model integrity and geometry after sterilization, this research has contributed to the broader understanding of the use of 3D-printed models for tailor-made endovascular solutions. As medical technologies continue to evolve, research endeavors such as this one can serve as a foundation for harnessing the full potential of 3D printing to advance patient-centered care in aortic surgery.

## 1. Introduction

Over the past decades, significant technological advancements in the endovascular surgery have revolutionized the options available to vascular surgeons, allowing them to offer modern therapeutic solutions for a variety of vascular conditions [1]. As a result, (thoracic) endovascular aortic repair ((T)EVAR) has emerged as the preferred primary method for treating most aortic disorders [2,3].

Managing complex aortic pathologies, defined as aneurysms or penetrating aortic ulcers (PAUs) that include the renovisceral segment, presents a particular challenge [4]. The use of fenestrated and branched stent grafts during complex endovascular aortic procedures has been shown to be a safe and viable treatment option in patients with complex abdominal aortic aneurysm (AAA) or thoracoabdominal aortic aneurysm (TAAA) [5]. Multicenter experiences and systematic reviews have consistently demonstrated high technical success rates and lower mortality and morbidity in the short-term when compared to traditional open surgical repair [6,7]. Endovascular therapy has proven to be particularly crucial in the management of acute and ruptured aortic pathologies.

Symptomatic aortic aneurysms with an impending rupture require timely surgical or endovascular therapy. Open surgical treatment is associated with high perioperative morbidity, leading to an increasing focus on endovascular treatment [8,9]. However, in the case of complex aortic pathologies, the adjacent organ arteries must also be treated or secured to ensure perfusion of vital organs. Nevertheless, the customization process for these fenestrated stent grafts is time-consuming, typically taking approximately 4 to 6 weeks [10]. Thus, this option hardly seems appropriate for patients with symptomatic complex aortic aneurysms.

This concern has led to the development of off-the-shelf fenestrated/branched stent-grafts (OSFGs) for most common anatomic variations of the aorta [6,11]. Despite this development, such OSFGs are not suitable for a large number of patients.

Therefore, the need for an alternative method has arisen, involving the utilization of available conventional stents with modifications made by surgeons for the treatment of acute patients [12]. An essential part of the manufacturing process of a surgeon-modified stent graft (SMSG) involves detailed planning of the positioning of the target vessel ostia on the SMSG to ensure their perfusion following the endovascular procedure [13].

Utilizing three-dimensional (3D) aortic templates is anticipated to elevate the precision and effectiveness of SMSGs as an integral component of personalized medicine. This strategy tailors interventions with exceptional accuracy to address distinct vascular issues specific to each patient [14]. In the context of a sterile operating room, surgeons integrate the aortic 3D template with a standard aortic stent graft, enabling precise marking of fenestration locations [15].

The use of 3D printing to guide the creation of fenestrations in SMSG for the treatment of complex AAAs or TAAAs has recently gained significance in an increasing number of vascular centers [14,16]. A sterile 3D model of the diseased section of the aorta allows surgeons to accurately mark the position of important arterial branches on the stent graft and incorporate corresponding openings on the stent graft itself before the SMSG is implanted [17,18].

The main questions that have arisen during the establishment of this method concern the standardization of the process—segmentation and preparation of the aortic portion of interest for 3D printing, as well as the sterilization and durability of the 3D aortic model after sterilization.

## 2. Materials and Methods

To create an accurate 3D digital aortic model, we used patient-specific aortic anatomical data acquired from computed tomography angiography (CTA) scans. The DICOM data from such a scan were segmented with computer-aided design (CAD) software to delineate the aortic geometry. The generated stereolithography (STL) file was transferred to a 3D printer to produce a patient-specific aortic resin model via additive manufacturing using computer-aided manufacturing (CAM) technology. 

**Figure 1:**
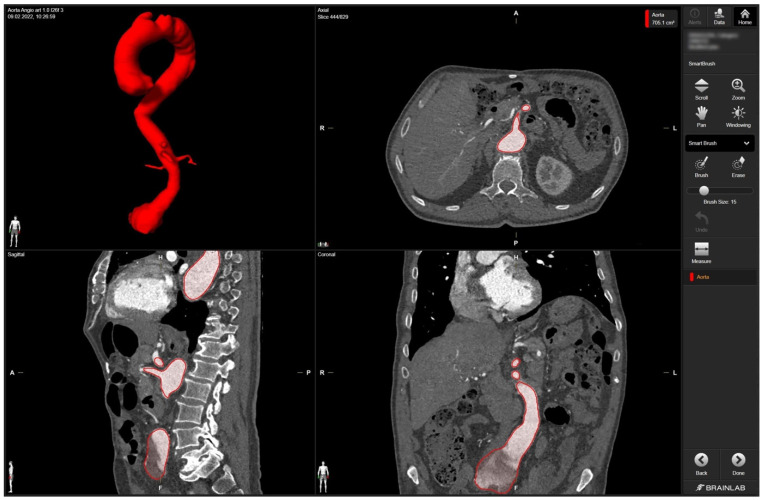
**Screenshot of 3D aortic model after the segmentation process in Brainlab Elements (backbone release 1.5.0.208) using the “smart brush” tool**. **Upper left corner**—fully sequenced aorta highlighted in red. In the **upper right corner**—axial view of the aorta marked in red with the mesenteric artery. In the **lower left corner**—sagittal view of the aorta with the initial portion of the mesenteric artery and part of the celiac trunk marked in red. In the **lower right corner**—coronary view of the aorta with the aortic segments IV and V marked in red.

In detail, radiologic data sets for the patient’s thoracic and abdominal regions (soft-tissue window with contrast medium at arterial phase, axial sections, 1-mm slice thickness) were exported from the hospital‘s PACS server in DICOM format. The data set was imported into Brainlab Elements (backbone release 1.5.0.208, Brainlab AG, Munich, Germany). After aligning the data set, segmentation was performed using the “smart brush” software tool. This tool allows semi-automatic, three-dimensional object segmentation, which is conducted after manual segmentation of the desired object in two perpendicular planes; the result is calculated within seconds and can be reviewed and edited by the observer to ensure faultless segmentation without cutouts, especially near the vascular wall. Segmentation of the complete aorta (ascending portion, arch, descending portion including juxtarenal aortic aneurysm) including the proximal few centimeters of the main abdominal branches (celiac trunk, superior mesenteric artery, right and left renal arteries) could be conducted following the contrasted inner vascular lumen (Figure 1).

**Figure 2:**
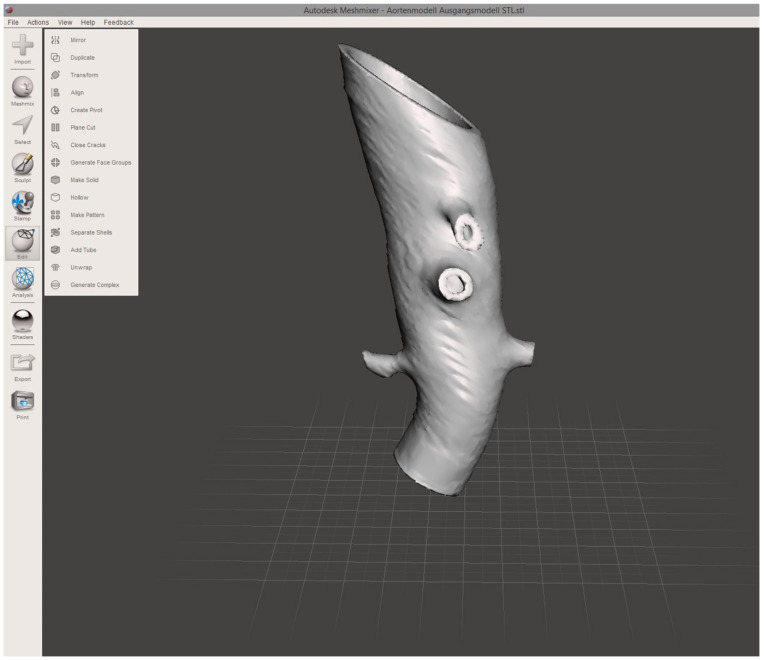
**Screenshot of finalized 3D aortic model.** The screenshot of the finalized 3D aortic model includes the outlets of the reno-visceral arteries (celiac trunk, superior mesenteric artery, right and left renal artery) in MeshMixer. The model serves as the basis for 3D printing.

To establish a model of the aortic wall, a second object was constructed by copying the segmented model and adding a centrifugal margin of 2.5 mm with the “safety margin” tool. By subtracting the first from the second model, the final 3D object representing the aortic vascular wall with a hollow intravascular lumen was built. The final 3D object was exported in STL file format at the high-quality setting. STL editing for 3D printing was performed using the freeware Autodesk Meshmixer (v3.5, Autodesk Research, San Francisco, CA, USA). Using the “transform” function, the 3D object was cut to reduce the model to the desired region of the first abdominal portion of the descending aorta, including the main abdominal branches (Figure 2). Final polishing and fixing of holes in the STL mesh was performed with the “inspector” software function to obtain a manifold model ready for 3D printing.

A biocompatible and sterilizable thermoplastic material (surgical guide resin V1; Formlabs Inc., Somerville, MA, USA) was chosen for printing the aortic models. The 3D printing was performed using the SLA printer Form 2 from Formlabs (Formlabs Inc., Somerville, MA, USA). The STL file for 3D printing was imported into the PreForm software (version 3.27.1, Formlabs Inc., Somerville, MA, USA) choosing the surgical guide resin V1 (Formlabs Inc., Somerville, MA, USA) and a slice thickness of 0.05 mm. Support structures were calculated automatically and manually edited to remove any support structures near the vascular outlet of the main aortic branches. The final print job was uploaded to the 3D printer and started. After printing was finished, the aortic model was post-processed according to the manufacturer’s protocol (Formlabs Inc., Somerville, MA, USA). Using the “form wash” function, the model was washed in 99% isopropyl alcohol for 20 min, air-dried for 30 min, and cured in the “form cure” function at 60 °C for 30 min. Further post-processing included the removal of the support structures and polishing at the sites of the removed support structures.

Five aortic 3D-printed models were post-processed according to the manufacturer’s protocol (Formlabs Inc., Somerville, MA, USA). Two models were subjected to sterilization and two models to disinfection using commonly employed methods. Another model remained untreated as a control sample.

For sterilization, the 3D-printed models were subjected to autoclaving, according to established protocols. The models were enclosed in sterilization pouches and exposed to standard autoclave conditions, including exposure to a temperature of 135 °C and a pressure of 3.116 mbar for 5 min. After autoclaving, the models were allowed to cool before further analysis. For disinfection, the subjected models were exposed to a maximum temperature of 95.9 °C using Neodisher^®^ Mediclean Forte Universal Cleaner (Dr. Weigert, Hamburg, Germany). All models were checked by in-house quality control for deformation (heat map analysis) following the sterilization or disinfection process.

After sterilization and disinfection, the aortic models were sent to the Institute for Medical Microbiology and Hospital Hygiene at the University Hospital, Düsseldorf, Germany for a sterility control. 

One sterilized model, one disinfected model, and the untreated model were sent for ex-house (Lufthansa Technik, AG, Materials Technologies and Central Laboratory Services, Hamburg, Germany) evaluation of possible structural changes. The models were quantitatively analyzed using advanced imaging and measurement technologies. The 3D-printed aortic models were subjected to thorough analysis to assess changes in structural properties and material composition using scanning electron microscopy (SEM) coupled with energy-dispersive X-ray spectroscopy (EDX). SEM imaging was conducted to visualize surface morphology changes, while EDX was employed to identify any variations in elemental composition resulting from the sterilization processes.

### 2.1. In-House Quality Control of the Deformation and Sterilization Process

**Figure 3:**
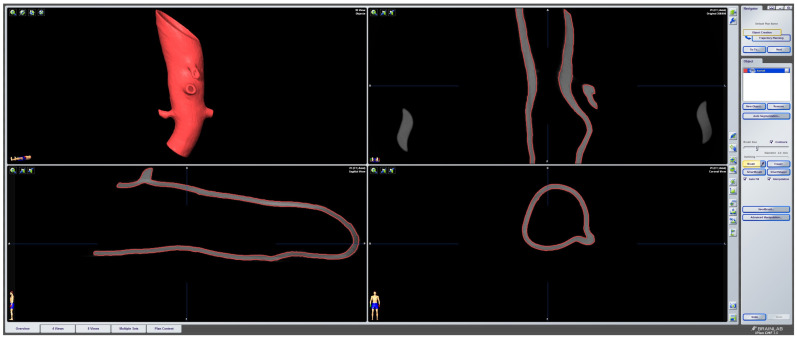
**Screenshot of pre-sterilized aortic model.** Screenshot of threshold-based segmentation of the pre-sterilized printed aortic model in iPlan CMF3.0 (version 3.0, Brainlab AG, Munich, Germany).

To evaluate the deformation of the CAD/CAM aortic model after the sterilization process, we performed a heatmap analysis. For this analysis, the final aortic model was scanned using a CBCT before and after the sterilization process. The data sets were imported into iPlan CMF (version 3.0, Brainlab AG, Munich, Germany) and a threshold-based segmentation of the aortic model was performed using an interval of Hounsfield units between −213 and + 2252 HU (Figure 3). The pre- and post-sterilization models were exported as STL files and imported into Geomagic Control X (software version 2020.1.1, Oqton, Los Angeles, CA, USA) for further analysis. The two imported STLs were aligned using the “best fit” option. The pre-sterilization model was defined as the reference data. A 3D heatmap analysis was performed using a range of +1/−1 mm for the color bar option and setting the tolerance at +0.5/−0.5 mm. A further detailed analysis of the strategically important areas of vessel outlets in the main branches was performed. For this analysis, measurements at four single coordinates circularly distributed along the surface mesh of the inner outlet of the four main branches (celiac trunk, superior mesenteric artery, right and left renal arteries) were performed three times. The mean and standard deviations of the absolute values of the measured discrepancies between the two meshes were calculated using Microsoft Excel (version 14.0, Microsoft Corporation, Washington, DC, USA).

As part of the clinic’s internal guidelines, the two aortic models were sent to the Institute of Medical Microbiology and Hospital Hygiene for a sterility check. Both models were incubated for 4 days at 36 °C under aerobic conditions in CASO-Boullion with lecithin, Tween 80, histidine, and thiosulfate (LTHth). CASO-Boullion is a trypticase soy medium that is routinely used for sterility testing as it supports the growth of a large variety of microorganisms (bacteria, yeasts, fungi). LTHth is added as standard to counteract the negative effect of residual disinfectant. At the end of the incubation period, no growth of microorganisms could be detected.

### 2.2. Ex-House Quality Control of the Deformation and Sterilization Process

#### Macroscopic Examination and SEM/EDX Analysis

**Figure 4:**
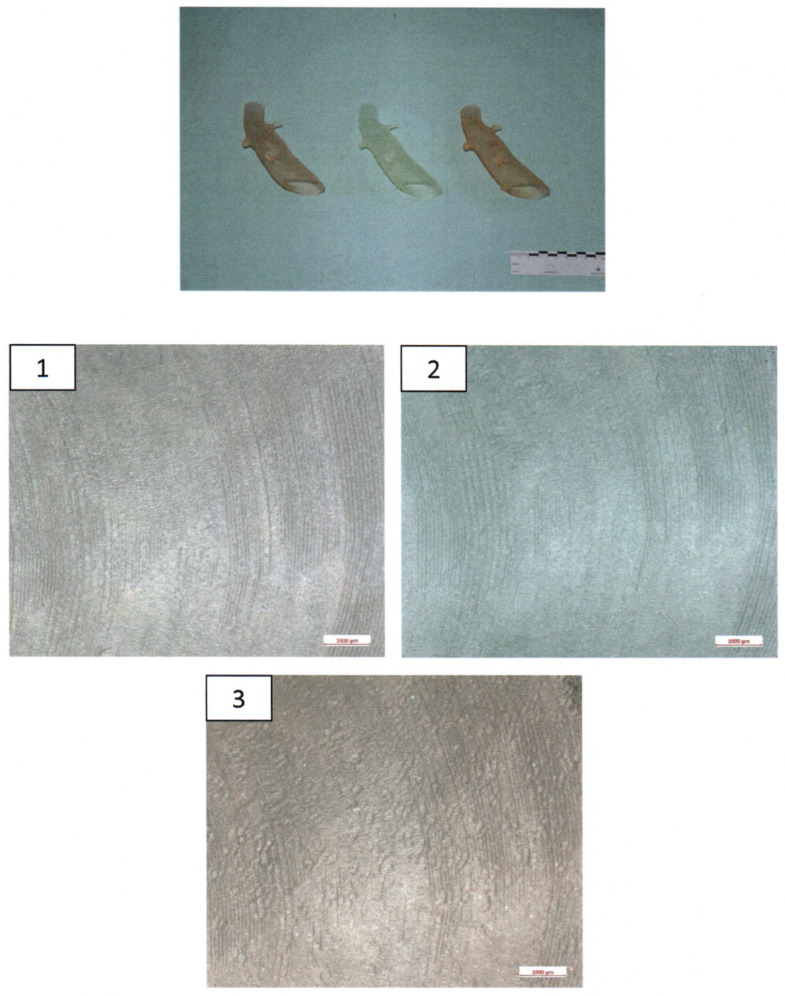
**Ex-house macroscopic examination of three aortic 3D Models.** Image on the **top**—three aortic models (**left**: model after disinfection, **middle**: model after sterilization, **right**: untreated model); inspection at magnifications of up to 30×—**1**.—model after disinfection; **2**.—Model after sterilization; **3**.—Untreated model.

The 3D-printed aortic models were subjected to thorough analysis to assess changes in structural properties and material composition using scanning electron microscopy (SEM) coupled with energy-dispersive X-ray spectroscopy (EDX). The surfaces of the implants were visually inspected at magnifications of up to 30× (Figure 4).

**Figure 5:**
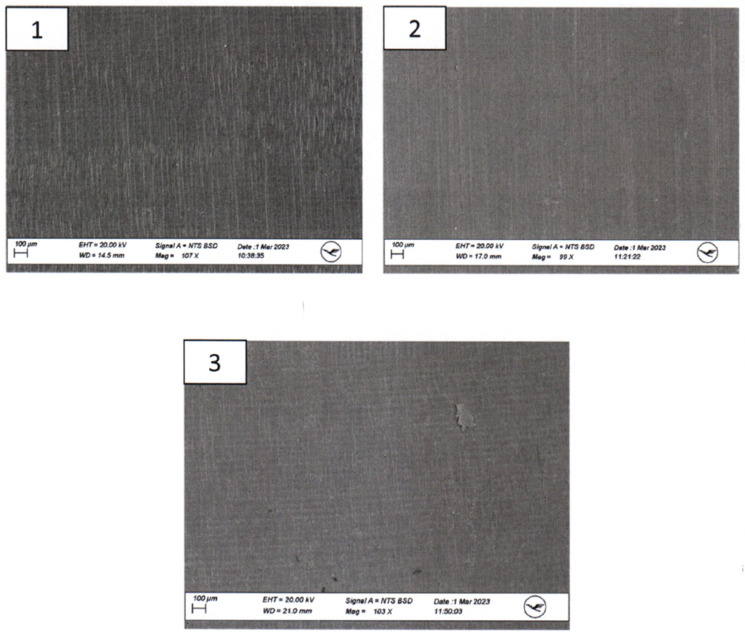
**Scanning Electron Microscopy (SEM) Examination of the proximal parts of the models. 1**.—model after disinfection; **2**.—model after sterilization; **3**.—untreated model.

SEM imaging was conducted to visualize surface morphology changes, while EDX was employed to identify any elemental composition variations resulting from the sterilization processes. For this segment of the investigation, a sample from the same area at the head end (smaller diameter) of each implant was extracted and coated with a layer of gold. All three implants exhibited a certain level of porosity exclusively in the upper edge region (Figure 5).

## 3. Results

### 3.1. Timeline of CAD/CAM Manufacturing

After clinical emergency assessment and performance of the indicated CT scan, the manufacturing process of the CAD/CAM aortic model could begin immediately. The entire procedure of computer-assisted design, including the steps of DICOM data export, segmentation, and editing of the 3D model, was completed in less than 20 min. 3D printing required 6 h and 54 min, with 1909 layers of 0.05 mm thickness printed. Post-processing required 90 min to produce the final 3D aortic model. The sterilization process took 71 min and 41 s, and the disinfection process took 47 min and 17 s. The entire procedure, from indication to surgery, required no more than 12 h.

### 3.2. In-House Analysis

#### 3D Heat Map Analysis of CAD/CAM Aortic Models

**Figure 6:**
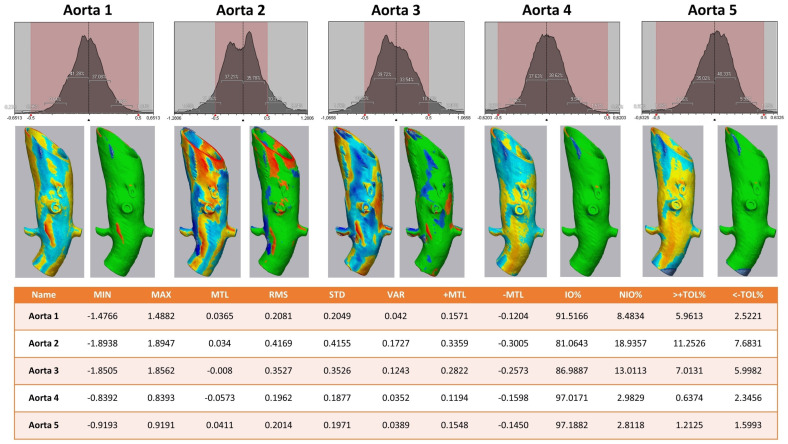
**Heat map analysis of five aortic models comparing different sterilization methods.** Aortic Models 1 and 4 were disinfected, 2 and 5 sterilized, and model 3 was untreated. **Upper row**: Value distribution of the 3D comparison showing the amount of STL triangles with a certain discrepancy between the pre- and post-sterilized surface meshes: the red area shows the tolerance interval of −0.5/+0.5 mm. **Middle row**: Heat map for each aortic model—on the left a continuous color map from under-contouring in dark and light red over yellow showing no discrepancy to light and dark blue indicating over-contouring; on the right side all values within the tolerance of −0.5/+0.5 mm are colored green. **Lower row**: Values of 3D comparison for each aortic model—MIN for minimum value for under-contouring, MTL for total mean value, MAX for maximum value of over-contouring, RMS for root mean square, STD for standard deviation, VAR for variance, IO% for percentage of values inside the tolerance interval, NIO% for percentage of values outside the tolerance interval, >+TOL% and <−TOL% for percentage of values above or below the tolerance interval, respectively.

The 3D comparison of the five different aortic models showed a stable form consistency with a root mean square of no less than 0.5 for each model after the sterilization process (Figure 6). The highest value of RMS reflected aortic model #2, with 81.1% of values within the set tolerance interval of −0.5/+0.5 mm.

**Figure 7:**
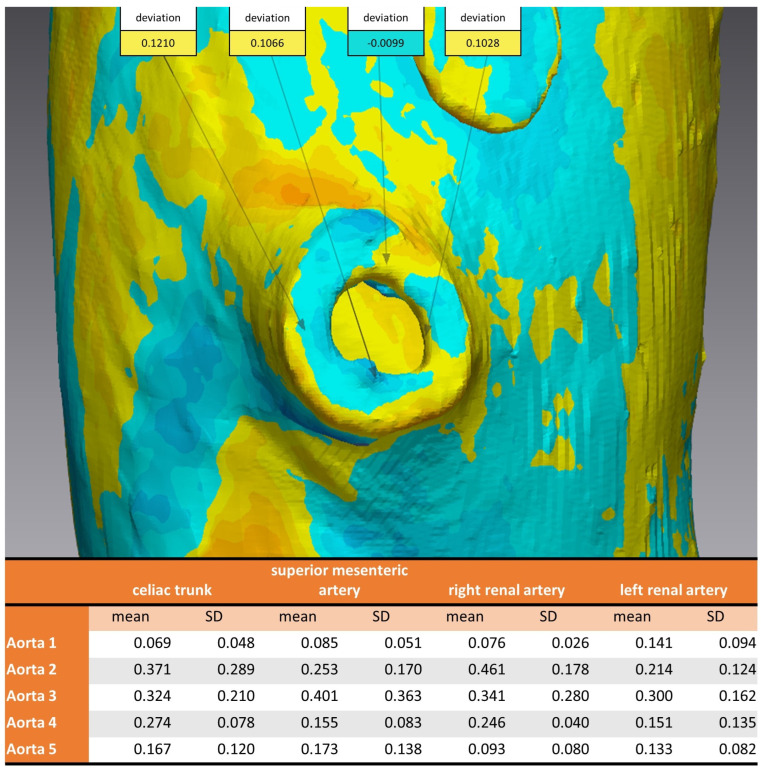
Analysis of 3D comparison at vascular outlet for different sterilization methods. Aortic Models 1 and 4 were disinfected, 2 and 5 sterilized, and model 3 was untreated. **Top:** example of measurements of four coordinates circumferential at the vascular outlet of the superior mesenteric artery. **Bottom:** mean and standard deviation for each aortic model at the vascular outlets of the four main branches of the abdominal aorta.

Considering the strategically important regions of the vascular outlets of the main branches of the abdominal aorta, the evaluation showed an overall mean discrepancy between pre- and post-sterilization of 0.2214 mm, and no value for mean discrepancy exceeded 0.5 mm (Figure 7).

### 3.3. Ex-House Analysis

The surfaces of the implants were visually inspected at magnifications of up to 30× (Figure 4). Implant #3 (untreated model) exhibited an additional irregular surface structure, approximately 1 cm^2^ in size, adjacent to the arc-shaped grooves present in all three implants. This particular surface structure was not observable in the corresponding areas of implants #1 (disinfected) and #2 (sterilized). No other distinguishing anomalies were identified on the implants.

**Figure 8:**
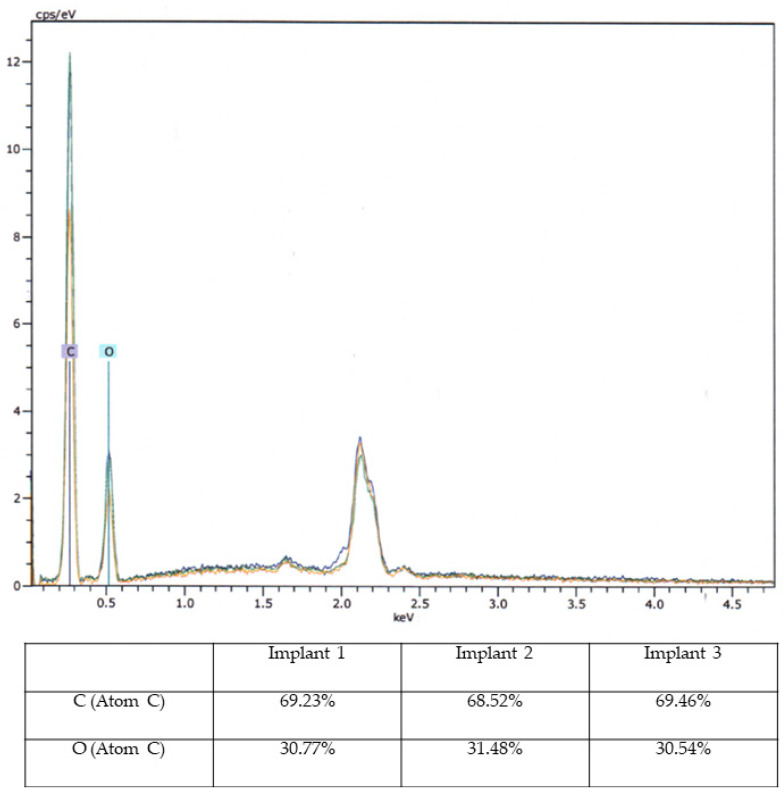
**Energy-Dispersive X-ray Spectroscopy Analysis.** Energy-Dispersive X-ray Spectroscopy (EDX) analysis of three aortic models with elemental composition (C and O) in diagram (upper image) and percentage table.

Based on macroscopic and SEM/EDX examinations, distinct evidence of material alterations attributed to a treatment process, such as a cleaning procedure, was not identified in the three implants. The comparative material analyses conducted via the EDX technique also yielded consistent results for all three implants (Figure 8).

### 3.4. Evaluation of the Sterilization Process

No microorganisms (bacteria, yeasts, fungi) were detected on the disinfected and sterilized models during the sterility test. 

## 4. Discussion

The present study focused on evaluating the suitability of 3D-printed aortic models for the development of SMSGs, with a specific emphasis on sequencing the aortic portion of interest and examining the impact of sterilization processes and potential material changes. The results of this investigation have provided valuable insights into the practical application of these models in the realm of vascular surgery.

Successful implementation of the described CAD/CAM (computer-aided design/computer-aided manufacturing) workflow described here for 3D-printed aortic models hinges on several critical factors, each of which contributes to the overall feasibility and effectiveness of this approach. This discussion presents the various requirements and considerations associated with arranging the proposed workflow. 

A fundamental requirement for the CAD/CAM process is the availability of personnel with the necessary expertise in computer-assisted surgery and CAD/CAM techniques. This expertise extends to both the creation of accurate 3D digital models and their translation into printable STL files. In addition, a competent team proficient in using the specific software applications associated with 3D modeling and printing is essential. Given the time-sensitive nature of certain surgical interventions, particularly in emergency settings, the availability of skilled staff capable of swiftly and accurately employing CAD/CAM methodologies becomes paramount. 

The CAD/CAM workflow necessitates the use of specialized software applications tailored for 3D modeling and printing. These applications enable the conversion of patient-specific anatomical data into digital models and, subsequently, into printable files. However, the acquisition of such software may require a significant financial investment to purchase the expensive software licenses. Additionally, successful implementation often involves collaboration with other groups such as cranio-maxillofacial (CMF), neurosurgery, and orthopedics departments, particularly in larger medical centers. Alternatively, freeware solutions such as 3D Slicer for Brainlab, Meshmixer, and CloudCompare for Geomagic Control X provide cost-effective alternatives without compromising functionality.

The adoption of 3D printing as a critical element of the workflow requires the availability of suitable hardware, specifically a 3D printer. However, the current landscape offers a range of 3D printing options that have become increasingly affordable and accessible. Additionally, the maintenance of modern 3D printers is relatively straightforward, minimizing disruptions to the workflow.

Macroscopic examination of the implants’ surfaces aimed to identify any apparent irregularities or changes in surface structures. Furthermore, SEM/EDX analysis was employed to examine the material composition of these implants. The observed porosity, primarily in the upper edge regions, was consistent across all three implants.

Examination of the implants following sterilization procedures was performed to ascertain whether the applied treatments had affected the implants’ structural integrity. Although sterilization methods are crucial for ensuring patient safety and device compatibility, they can potentially influence material properties. Importantly, the SEM/EDX analysis indicated that the sterilization methods did not lead to overt material changes or structural disruptions. This observation supports the premise that these 3D-printed aortic models can withstand sterilization processes without compromising their integrity, aligning with their potential use in the development of surgeon-modified endoprostheses.

In 2019, Rynio et al. conducted a study in which they outlined the fabrication of a 3D-printed aortic arch template using six commonly employed printing materials, namely polylactic acid (PLA), nylon, polypropylene (PP), polyethylene terephthalate glycol (PETG), and both rigid and flexible photopolymer resins [15]. This was achieved by using fused deposition modeling (FDM) and stereolithography (SLA) techniques. The 3D aortic models underwent sterilization through various methods. Notably, one of these methods involved autoclaving at 121 °C, and it was observed that steam sterilization in an autoclave at 121 °C induced significant deformation in the aortic templates constructed from PLA, PETG, and PP. Conversely, the remaining materials exhibited stable geometries. Moreover, during mesh comparisons, changes were found to be submillimeter in magnitude, a finding consistent of our study with dental guide resin.

From the existing literature, transparent and rigid resins have emerged as the preferred materials within the scope of current technological capabilities. This preference is rooted in their ability to offer clear visibility of rings or struts during the process of fenestration planning. Nonetheless, the inherent rigidity of these materials fails to accommodate the influence of guidewires on the straightening of the aorta and iliac arteries, a factor that extends to the final deployed endograft. Consequently, while the integration of 3D-printed templates holds promise for enhancing the precision of fenestration placement, it is important to acknowledge that the template’s rigidity does not entirely mitigate the potential for ostial mismatch [19].

Certain studies have demonstrated favorable clinical outcomes following the implantation of SMSG using 3D aortic models [14,16]. From this information, it can be concluded that the successful incorporation of 3D printing can be seamlessly integrated into the daily practice of physicians involved in stent graft modification. This is especially applicable to acute thoracoabdominal aortic pathologies requiring urgent intervention. As mentioned in the introduction, custom-made prostheses are necessary for managing such pathologies, with production typically requiring several weeks. However, after refining the sequencing, production, and sterilization processes, it is feasible to have a sterilized aortic 3D model within 10–12 h, enabling surgeons to intraoperatively create a personalized aortic prosthesis tailored to the patient from the tube prosthesis. Naturally, material and technical requirements (such as computers, software, and 3D printers) and trained medical personnel are essential for the entire process. From our experience, the assistance of additional personnel, such as IT engineers, is not necessary after the surgeon has received software training provided by the manufacturer.

It is important to acknowledge the limitations of this study. Its focus was primarily on macroscopic and microscopic analyses of the implants’ surfaces and material composition. In-depth mechanical testing and long-term durability studies could provide additional insights into the implants’ stability.

## 5. Conclusions

This study has contributed to the growing body of knowledge concerning the practical use of 3D-printed aortic models in vascular surgery. The absence of significant material changes following sterilization treatments indicates their potential suitability for the development of surgeon-modified endoprostheses. The findings also emphasize the importance of understanding the nuances of material properties and responses to various processes. As advancements in both 3D printing technology and material science continue, these models hold promise for enhancing the precision and customization of endovascular interventions, ultimately translating to improved patient outcomes. Further research, including extended clinical trials, could provide a more comprehensive understanding of the applicability of 3D-printed aortic models in surgical practice.

## Data Availability

The underlying data are available from the corresponding author upon reasonable request.
